# Inflatable Penile Prosthesis Complication in the Emergency Department

**DOI:** 10.3390/healthcare12100964

**Published:** 2024-05-08

**Authors:** Farzad Sedaghat, Aws Kamona

**Affiliations:** The Russell H. Morgan Department of Radiology and Radiological Science, Johns Hopkins University School of Medicine, Baltimore, MD 21287, USA; akamona1@jhu.edu

**Keywords:** inflatable penile prosthesis, erectile dysfunction, urethral perforation, urologic complication, emergency radiology, CT scan, 3D volumetric rendering

## Abstract

Inflatable penile prostheses are a widely utilized treatment for erectile dysfunction. While MRI is the optimal imaging modality for patients with suspected implant complications, it is often unavailable in the acute setting. In light of these limitations, we present a case of urethral perforation by an implanted penile cylinder and its evaluation with contrast-enhanced computed tomography (CT) in an emergent setting.

## 1. Introduction

Erectile dysfunction (ED) is defined as the persistent inability to achieve or maintain an erection sufficient for satisfactory sexual performance. It is a multifactorial condition, increasing in prevalence with advancing population age, with an estimated 52% prevalence in men aged 40–70 years old. Notably, the most significant condition associated with ED is cardiovascular disease (CVD), which results in impaired vasodilation, resulting in insufficient penile blood flow and an inability to overcome venous outflow, resulting in ED. Other less common causes include hypogonadism, neurological disorders, such as spinal trauma and multiple sclerosis, psychological factors, side effects of medications, and anatomical disorders such as Peyronie’s disease [1].

The management of ED usually starts with proper clinical assessment, lifestyle changes, and modifying drug therapy that may cause ED. First- line pharmacotherapy is with oral phosphodiesterase type 5 inhibitors. If these treatments are not successful, vacuum constriction devices, intraurethral alprostadil, and intracavernous injection of a vasoactive drug are available as second-line treatments, followed by penile prostheses (PP) as a third-line treatment [2]. Inflatable penile prostheses are the most commonly implanted devices for medically refractory ED patients and for those with contraindications to pharmacotherapy [3].

Inflatable penile prostheses (IPPs) are mainly divided into two- and three-piece devices; both of which are composed of saline-containing cylinders inserted into each of the 2 corpora cavernosa. In the three-piece device, a pump inserted in the scrotum and a reservoir containing the saline fluid is usually inserted in the pelvis next to the bladder. In the two-piece device, the pump and reservoir are combined in one component inserted in the scrotum that transfers fluid from the proximal to distal parts of the cylinders. Another type of penile prosthesis, which is less commonly used and is not the main focus of this report, is the malleable or semi-rigid penile prosthesis (MPP), which is composed of two non-inflatable shafts implanted in each of the corpora cavernosa. They are less expensive; however, they have a higher risk of lateral perforation and distal erosion, as well as the discomfort and embarrassment for the patient from the permanent erection caused by these rigid implants. Nonetheless, they are still utilized in patients for whom inflatable prostheses are not a viable option, including those with spinal cord injury, who have impaired manual dexterity, and pelvic organ transplant recipients, who are at increased risk of infection [4].

Imaging plays a vital role in the assessment of PPs, MRI is considered the modality of choice for assessment of penile prosthesis, MPP cylinders appear hypointense on T1 and T2 sequences. IPP cylinders appear hyperintense on T2 due to saline filling, while the extenders at the base of the penis and the silicone-based cylinder covering appear to be T2 hypointense; the pelvic reservoir normally appears as a T2 hyperintense (cystic) oval/round structure next to the urinary bladder with thin wall, and the scrotal pump placed in the subdartos pouch between the two testicles appears as a small oval T2 hyperintense structure, and the three components are connected by thin tubes that appear T2 hypointense. It is worth noting that in the deflated state, the IPP cylinders can appear buckled and deformed, and in general, an MRI assessment of the IPP should be performed with cylinders in an inflated state.

On CT, the IPP appears as fluid-filled cylinders with hyperdense extenders at the penile base, the perivesical reservoir and scrotal pump appear as fluid density oval/round structures with thin walls, and communicating thin dense tubing.

Other modalities have limited use in assessing PPs; for example, ultrasound, while used for preoperative diagnoses of ED, has less of a role in PP complication assessment, primarily limited to assessing the anechoic pelvic reservoir and scrotal pump, and potential associated abscesses. Radiography is considered obsolete for IPP complication assessment; however, can be used to assess MPPs for fractures [2].

The reported overall penile prosthesis survival at 5 and 10 years is 83% and 78–82%, respectively [5]. Erosion is a rare complication that occurs in less than 1% of patients and is most commonly seen with non-inflatable implants or in association with device infection [4].

We present a case of IPP erosion into the corpus spongiosum many years after implantation and review its diagnosis and management in an emergent setting.

## 2. Case Report

A 75-year-old man with a past medical history of benign prostatic hyperplasia, Peyronie’s disease, coronary artery disease, type 2 diabetes mellitus (DM), hypertension, and hyperlipidemia presented to our emergency department with concern for erosion of his implantable penile prosthesis, which was implanted 7 years ago in another institution.

The patient’s symptoms started a month before presentation, shortly after a large dog jumped on his groin. Since then, he noticed pain and feeling of the prosthesis more superficially. This was followed by noticing spraying of his urine stream. The patient did not seek medical care at this point; however, one day before presenting to our emergency department, the patient noticed the device protruding from his urethra and saw his outpatient urologist, who advised him to go to the emergency department.

**Figure 1:**
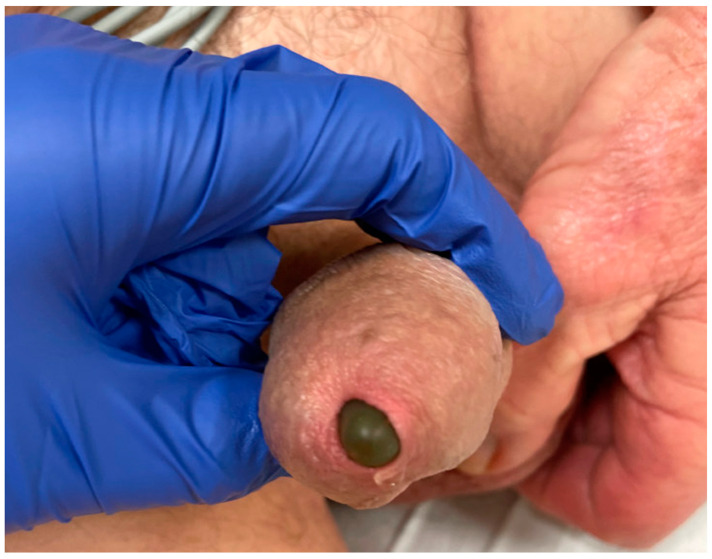
A picture demonstrating the perforating cylinder protruding through the urethral meatus.

**Figure 2:**
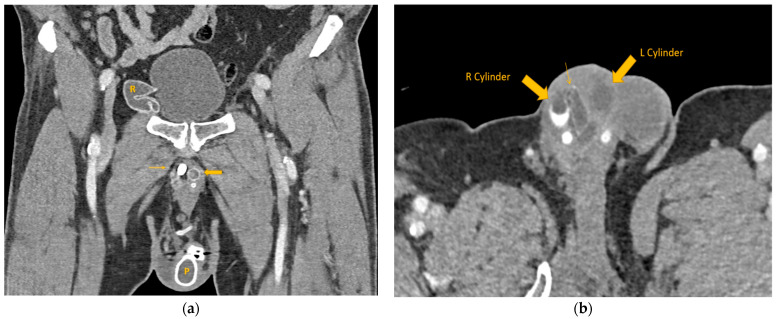
Contrast-enhanced CT of the abdomen and pelvis. (**a**) Coronal reformation demonstrating the decompressed reservoir in the right hemi pelvis adjacent to the bladder, and the pump noted in the left scrotum. The left fluid-filled cylinder (broad arrow) is appropriately positioned in the left corpus cavernosum. At the same level, the metallic rear tip extender is seen in the right corpus cavernosum (thin arrow) due to anterior migration of the right cylinder. (**b**) Axial image focused on the proximal penis, demonstrating the right and left cylinders with focal kinking of the proximal right cylinder (thin arrow) and central inferior extension of the cylinder, at the site of suspected urethral erosion. R: reservoir, P: pump.

**Figure 3:**
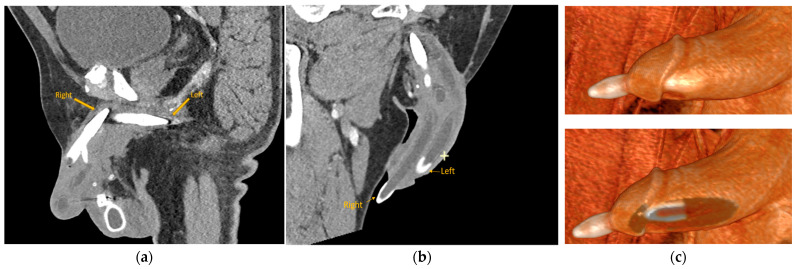
Contrast-enhanced CT of the abdomen and pelvis. (**a**) Sagittal reformation demonstrating the anterior displacement of the right metallic rear tip extender and the normal position of the left rear tip extender. (**b**) Oblique reformation of the penis demonstrating the transurethral extension of the right penile cylinder. (**c**) Volumetric 3D rendering of the penile prosthesis demonstrating the abnormal protrusion of the right penile prosthesis cylinder through the meatus, and left cylinder in the expected location.

At the time of presentation in the emergency department, the patient had stable vital signs and reported no dysuria, fever, chills, or abdominal pain. The urinalysis and routine laboratory work-up came back within normal limits, without signs of urinary tract infection. On physical examination, a straight circumcised phallus was noted with protrusion of the gray device tip at the urethral meatus (Figure 1), no associated erythema or drainage from the meatus was observed. The pump noted in the scrotum was hard and could not be cycled at bedside. A standard portal venous phase contrast-enhanced CT was performed using a 128-slice helical scanner (SOMATOM Definition Flash 128 slice helical CT scanner (Siemens Healthineers, Forchheim, Germany), institutional protocol detailed at http://www.ctisus.com/protocols, accessed on 1 May 2024), demonstrating erosion of the right cylinder into the penile urethra with protrusion through the urethral meatus (Figure 2). Post-acquisition processing with 3D volumetric rendering techniques using Syngo.via (Siemens Healthcare, Erlangen, Germany) was performed on the reading station (Figure 3).

The patient was administered antibiotics and underwent complete device extraction 2 days after presentation to the emergency department. No large urethral defect was cystoscopically apparent following device removal. A Foley catheter was then placed for 2 days to allow the urethral defect to heal. After its removal, the patient experienced no difficulty voiding and was discharged without complication. The patient was seen for a follow-up several months later and was in his usual state of health. He inquired regarding options for ED, and was advised that he would be at high risk for repeat erosion and infection; moreover, they may be dissatisfied with the IPP, as corporal excavation could be required, leading to reduced girth. Given these risks, the patient deferred repeat IPP placement.

## 3. Discussion

Complications of penile prosthesis (PP) can be broadly divided into three categories:

### 3.1. Mechanical Failure

Considered the most common cause for revision surgery for patients with PP, fractures are more common with malleable prosthesis and usually preceded by trauma; aneurysm, a unique complication of IPP presenting with the focal bulge of the cylinder that disappears with deflation, can happen due to wear and tear or due to a manufacturing defect; leakage, another unique complication in IPP due to rupture or leakage of saline from one of the saline containing cylinders, which could be caused by trauma or a manufacturing defect, usually presenting with nonfunction or insufficient inflation of device).

### 3.2. Malpositioning

Inappropriate cylinder size results in buckling with the use of oversized cylinders and floppy glans with the use of a too-short cylinder, erosion and migration, as discussed below, migration without erosion.

### 3.3. Infection

Infection is one of the most serious complications that often requires surgical intervention and PP removal. Risk factors include diabetes, trauma, and neurological deficits such as spinal cord injury. The reported infection rate ranges from 1% to 3% in low-risk patients. Infection is usually categorized into early infection (less than 6 weeks after PP surgery), usually caused by gram-negative bacteria and can be diagnosed clinically, typically presenting with infectious symptoms such as fever, erythema, edema and purulent discharge, and imaging is rarely needed; and late infection (6 weeks or more after the PP surgery), typically caused by opportunistic bacteria with subclinical presentation, often presenting with pain that increases with erection, which can involve only a prosthetic component, and imaging usually with MRI is often needed to look for the extent of infection, presence of small collections, and soft tissue involvement, which can alter management [5].

Less frequent complications of the reservoir can occur, including rupture and fluid leakage, which usually presents clinically with complete failure of erection or inadequate rigidity after inflation; reservoir migration, usually asymptomatic and incidentally discovered on imaging; and infection of the reservoir, which can result from spread of pelvic infection or rupture. Other very rare causes for reservoir dysfunction can be seen with fibrosis in the perivesical space from prior surgery or radiation.

The least commonly reported complications are complications involving the scrotal pump, which include infection, hematoma formation, and migration [5].

Urethral perforation is a known rare complication that occurs in approximately 1% of penile prosthesis surgeries and is more commonly seen in patients with corporal fibrosis, including diabetics, post-infectious fibrosis, and Pyronie’s disease. Most perforations occur intraoperatively during corporal dilation and bending; hence, multiple techniques and tools were invented to reduce such risks; however, the description of these techniques is beyond the scope of this case report.

Delayed perforation is described in the context of subtle missed intraoperative distal urethral injury, which leads to a fistulous connection and urethral perforation over time. Delayed perforation may also be seen in patients with frequent urethral instrumentation (which leads to chronic friction and cylinder erosion into the urethra) recurrent infection, and compromised distal penile sensation, (seen in paraplegics, diabetics, and irradiated patients) [6].

Although most complications are diagnosed clinically, imaging is critical in subtle and equivocal cases. MRI is the preferred modality for evaluation of penile prostheses because of its higher soft tissue resolution, absence of ionizing radiation, better assessment of the penile anatomy, and superior evaluation of the prosthesis components [4]. However, MRI is not readily available in emergency settings to some centers and requires longer acquisition times, which might not be suitable for unstable patients. CT remains the work horse in emergency settings due to availability and fast acquisition of images, and radiation is not usually a major concern in IPP patients due to advanced age. Most published literature is focused on MRI assessment of penile prostheses, and only a few studies describe the role of CT. For example, one study demonstrated CT findings reliably predicted subsequent postoperative findings in 33 patients with IPP complications [7].

In our case, the perforation occurred 7 years after the prosthesis implantation in a patient with risk factors including Peyronie’s disease and DM. Notably, the precipitating insult may have been the traumatic injury described by the patient, when his heavy dog jumped on his lap, causing a minor urethral injury that progressed to a cavernourethral fistula over the monthlong period.

CT with intravenous contrast correlated well with the operative findings and was sufficient for preoperative assessment and planning, with high-fidelity 3D reconstructions obviating the need for MRI. To the best of our knowledge, this is the first published case of penile prosthesis perforation demonstrated by contrast-enhanced CT.

## 4. Conclusions

IPP erosion is a rare complication that can be adequately assessed using contrast-enhanced CT without the need for a subsequent preoperative MRI. Knowledge of the device components and appearance on CT is important for the detection of complications in the emergency setting.

## Data Availability

Data is contained within the article.
